# Acute Dehydration Impairs Endurance Without Modulating Neuromuscular Function

**DOI:** 10.3389/fphys.2018.01562

**Published:** 2018-11-02

**Authors:** Oliver R. Barley, Dale W. Chapman, Anthony J. Blazevich, Chris R. Abbiss

**Affiliations:** Centre for Exercise and Sports Science Research, School of Medical and Health Sciences, Edith Cowan University, Joondalup, WA, Australia

**Keywords:** combat sports, dehydration, hypohydration, weight cutting, weight loss

## Abstract

**Introduction/Purpose:** This study examined the influence of acute dehydration on neuromuscular function.

**Methods:** On separate days, combat sports athletes experienced in acute dehydration practices (*n* = 14) completed a 3 h passive heating intervention (40°C, 63% relative humidity) to induce dehydration (DHY) or a thermoneutral euhydration control (25°C, 50% relative humidity: CON). In the ensuing 3 h *ad libitum* fluid and food intake was allowed, after which participants performed fatiguing exercise consisting of repeated unilateral knee extensions at 85% of their maximal voluntary isometric contraction (MVIC) torque until task failure. Both before and after the fatiguing protocol participants performed six MVICs during which measures of central and peripheral neuromuscular function were made. Urine and whole blood samples to assess urine specific gravity, urine osmolality, haematocrit and serum osmolality were collected before, immediately and 3 h after intervention.

**Results:** Body mass was reduced by 3.2 ± 1.1% immediately after DHY (*P* < 0.001) but recovered by 3 h. Urine and whole blood markers indicated dehydration immediately after DHY, although blood markers were not different to CON at 3 h. Participants completed 28% fewer knee extensions at 85% MVIC (*P* < 0.001, *g* = 0.775) and reported a greater perception of fatigue (*P* = 0.012) 3 h after DHY than CON despite peak torque results being unaffected. No between-condition differences were observed in central or peripheral indicators of neuromuscular function at any timepoint.

**Conclusion:** Results indicate that acute dehydration of 3.2% body mass followed by 3 h of recovery impairs muscular strength-endurance and increases fatigue perception without changes in markers of central or peripheral function. These findings suggest that altered fatigue perception underpins muscular performance decrements in recovery from acute dehydration.

## Introduction

Total body water is essential to the physiological function of the human body. As such, the balance of total body water has been the topic of much previous research, with inducing a deficit in total body water (dehydration) and the effects on exercise performance being a large focus (Horswill and Janas, 2011; Cheuvront and Kenefick, 2014). Dehydration of 2–3% of body mass (BM) has been found to impair both aerobic exercise performance, especially when convective cooling is minimal (Barr, 1999; Cheuvront et al., 2010), and anaerobic performance (Kraft et al., 2012). The research is less clear when examining ultra-endurance performance with studies finding increased dehydration to be associated with improved performance (Zouhal et al., 2010; Knechtle et al., 2012). The method of how dehydration is induced may also be of some importance, as it can be induced using exercise (actively) or by using environmental stress at rest (passively) (Cheuvront and Kenefick, 2014). The negative effects of both active and passive dehydration have also been found to persist even following rehydration, with researchers reporting impairments in repeat-effort capacity (Barley et al., 2017b), sports-specific skills (Baker et al., 2007), mood (Hall and Lane, 2001) and cognitive function (Choma et al., 1998) for up to 5 h, and in some cases 24 h, despite *ad libitum* rehydration. Such findings have substantial implications for athletes in sports with weight classifications (e.g., combat sports), as athletes often attempt to rapidly lose and regain body mass prior to being weighed for competition, with athletes reporting losing 3–5% of their body mass rapidly before being weighed for competition (Barley et al., 2017a).

Dehydration impairs exercise performance through multiple mechanisms including an increased cardiovascular strain (González-Alonso et al., 1997), a reduced muscle blood flow compromising oxygen delivery and aerobic metabolism (Cheuvront et al., 2010), impaired thermoregulatory function (Casa, 1999; Cheuvront et al., 2010), and increased carbohydrate metabolism (Casa, 1999). Dehydration may also compromise neuromuscular function as a result of alterations in electrolyte concentrations (particularly sodium and potassium) within the interstitial and intracellular spaces (Sjøgaard, 1985; Casa, 1999). Electrolyte balance is important for the maintenance of membrane electrochemical potential and actin-myosin function which, if significantly altered, may reduce function of neurones and muscle fibers (Sjøgaard, 1985). Consequently, dehydration may impair physical performance as a result of fatigue occurring both proximal (central) and distal (peripheral) to the neuromuscular junction (Minshull and James, 2012). Thermal exposure has also been found to negatively influence neuromuscular function both and rest and following exercise which may be of importance when thermal stress is used to induce dehydration (Ross et al., 2011; Goodall et al., 2015). Research examining the effects of dehydration on physical performance and neuromuscular function is mixed, with studies typically observing a negative (Bigard et al., 2001; Ftaiti et al., 2001; Minshull and James, 2012; Bowtell et al., 2013) or no effect (Evetovich et al., 2002). However, the effects of acute dehydration on performance and neuromuscular function following a periods of recovery and/or rehydration is even less clear due to a paucity of research (Bigard et al., 2001; Rodrigues et al., 2014). As a result, it is currently unclear whether the reduction in exercise capacity observed in the hours following dehydration (Hall and Lane, 2001; Barley et al., 2017b) results from prolonged alterations in neuromuscular function (i.e., central and peripheral fatigue) or other mechanisms such as altered carbohydrate metabolism or mental fatigue (Sjøgaard, 1985; Montain et al., 1998; Casa, 1999; Bigard et al., 2001; Minshull and James, 2012). Indeed, dehydration has also been found to negatively influence mood and cognitive function (Choma et al., 1998; Hall and Lane, 2001) and increase perceived exertion, which could be associated with increased mental fatigue (Marcora et al., 2009; Barley et al., 2017b). While it is possible that changes in mood and cognition are a consequence of changes in neural function, we are not aware of any studies that have examined this in detail. As such, the aim of the present study was to examine the influence of 3% acute dehydration on muscular force production and endurance, neuromuscular markers of central and peripheral fatigue, mood and cognition in combat sport athletes with experience in acute dehydration strategies. While previous research has investigated the influence of dehydration on neuromuscular function and muscular endurance, we are unaware of research that has conducted an in-depth evaluation of the potential central and peripheral changes in neuromuscular function following rehydration. We hypothesized that acute dehydration would impair muscular endurance alongside central and peripheral neuromuscular function.

## Materials and Methods

### Participants

Fourteen highly trained male combat sports athletes (age 25 ± 4 years, height 1.8 ± 0.05 m, body mass 80 ± 11 kg) with no history of leg injuries and at least 2 years of competitive combat sports experience volunteered for the study. Subjects were recruited via advertisements or word of mouth. All participants were required to have experience using acute dehydration strategies to make weight for competitions. We conducted an *a priori* power analysis using previous research investigating the reliability of neuromuscular assessments of the quadriceps muscles (Place et al., 2007) and estimated that 14 participants would be required to identify statistical differences of a 0.25 effect size or greater with 95% power and an α level of 0.05. This study was carried out in accordance with the recommendations of the Australian National Statement on Ethical Conduct in Human Research with written informed consent from all subjects. All subjects gave written informed consent in accordance with the Declaration of Helsinki. The protocol was approved by the Edith Cowan University Human Research Ethics Committee.

### Study Design

Participants completed one familiarization session and two experimental sessions. In the familiarization session, assessments of mood, cognition and neuromuscular function were practiced until the participants expressed confidence and provided repeatable results. The experimental sessions were performed at the same time of day and separated by at least 7 days, with the session order randomized and counterbalanced. During experimental sessions participants performed either a dehydration (DHY) or control (CON) protocol, which were both followed by 3 h of *ad libitum* food and fluid intake. DHY involved 3 h of passive heat exposure to induce dehydration which is a common method of weight loss in combat sports (Barley et al., 2017a) while CON involved 3 h of exposure to thermoneutral conditions as detailed below. Mood was assessed before, immediately and 3 h after both DHY and CON. Cognition and neuromuscular function were assessed only before and at 3 h after DHY and CON. Cognition and neuromuscular function were not assessed immediately following DHY and CON due to the likelihood of the testing influencing performance in the tests at 3 h. Each participant was asked to record their nutritional intake for the 24 h prior to and during the first experimental session and then replicate intake during the second experimental session.

DHY involved 3 h of passive heat exposure in an environmental chamber at 39.9 ± 0.3°C, and 63 ± 2% relative humidity with the aim of reducing body mass by 3–4%. Participants wore a plastic suit (plastic sweat suit, Wrap Yourself Slim, Australia) and were not permitted to consume fluids during the protocol. CON involved 3 h of thermoneutral exposure (23.5 ± 0.7°C, and 50 ± 12%) with the aim of maintaining body mass and euhydration. Participants were permitted to drink fluids throughout the protocol. Core temperature was continuously monitored throughout the experimental trials using a gastrointestinal pill ingested 4 h prior to testing (CorTemp ingestible core body temperature sensor, HQinc, United States). Heart rate was continuously recorded using a Polar heart rate monitor (Model S810i, Polar Electro Oy, Kempele, Finland). Following both DHY and CON participants were free to consume food and fluid *ad libitum*, with the participants encouraged to consume food and fluid as they would in preparation for a competition.

Immediately before, after and 3 h after DHY or CON, nude body mass (Mettler 1D1 multirange, FSE, Australia), brachial blood pressure (Automatic blood pressure monitor, OMRON, Singapore), core temperature, tympanic temperature (ThermoScan, Braun, Germany) and heart rate were recorded. At these same time points, finger prick whole blood samples were collected into capillary tubes (Capilette MPW-212, MPW med. Instruments, South Australia) and spun at 3600 rpm to determine haematocrit (Centrifuge MPW-212, MPW med. Instruments, South Australia), whilst urine samples were collected and assessed for osmolality (Advanced 3250 single-sample osmometer, Advanced instruments, United States) and urine specific gravity (Atago hand refractometer, model UNC-NE, Atago, Japan). Venous blood samples were collected into an 8.5 ml serum separating tube vacutainer and spun at 12000 rpm for 15 min at 4°C in a centrifuge (Multifuge 3 S-R, Kendro, United States) to obtain serum. A 200 μl sample of serum was assessed for osmolality (Advanced 3250 single-sample osmometer, Advanced instruments, United States) while the remaining serum was aliquoted equally and immediately frozen at -80°C to be later analyzed for brain-derived neurotropic factor (BDNF) to evaluate any potential physiological influences on cognition (Roh et al., 2017) and tumor necrosis factor alpha (TNFα) to evaluate any potential tissue damage resulting from heat stress (Collins and Grounds, 2001) using enzyme-linked immunosorbent assay kits (Quantikine HS ELISA, R&D Systems, Minneapolis, MN, United States).

### Profile of Mood States Short Form

Mood was assessed using a Profile of Mood States Short Form (POMS-SF). The POMS-SF assesses anger, confusion, depression, fatigue, tension and vigor. Anger items included “Angry” and “Annoyed”; confusion items included “Bewildered” and “Forgetful”; depression included terms such as “Unhappy” and “Hopeless”; fatigue items included “Worn out” and “Fatigued”; tension items included “On Edge” and “Nervous”; and vigor included terms such as “Active” and “Lively.” Items were rated on a 5-point scale ranging from “Not at all” [0] to “Extremely” [5]. Responses to the POMS-SF have been found to be comparable to the original POMS (Curran et al., 1995).

### Cognitive Assessment

Cognitive function was assessed using the CogState computerized test battery (CogState, CogState Ltd, Melbourne, VIC, Australia). Cogstate has been found to have a high test–retest reliability and to be sensitive to mild changes in cognitive state (Collie et al., 2003). The test takes approximately 15 min and utilizes playing cards as the stimulus. The test assesses simple reaction time, choice reaction time, attention/visual learning and memory, and attention and working memory. Response time and accuracy were reported for each task. In order to minimize potential learning effects, the participants were familiarized with the protocol during the familiarization session.

### Neuromuscular Assessments

Neuromuscular function was assessed using a maximal voluntary isometric knee extension contraction (MVIC) protocol before DHY or CON as well as both before and after a fatiguing knee extensor exercise protocol at 3 h after DHY and CON. The MVIC protocol required the performance of six maximal voluntary isometric contractions. Three were performed using brief contractions (<2 s) with the verbal instruction “as fast as possible” whilst another three required longer contractions (3–5 s) utilizing the verbal instruction “as hard and as fast as possible”; a 30-s rest was provided between efforts (Maffiuletti et al., 2016). An electrical stimulus was applied to the quadriceps during the three longer contractions to assess muscle voluntary activation, as described below. The participants were seated in an isokinetic dynamometer (Biodex System 3 Pro, Biodex Medical System, Shirley, NY, United States) with their dominant leg attached and the knee fixed to 60° (0°= Full extension). Before tests (as a warm-up) the participants performed eight brief voluntary knee extensor contractions beginning at 30% of perceived MVIC and progressively increasing until reaching 100% of perceived MVIC for the final contraction. A 2-min rest was given before the testing commenced. The contractile rate of force development (RFD) was assessed using the torque generated in the first 75 ms of each MVIC (T_75__ms_). This time epoch was chosen as it is reported that RFD is strongly influenced by neural factors in this time period (Maffiuletti et al., 2016). Additionally, the reliability of shorter time periods (i.e., 30–50 ms) may be poor when conducted on commercially available dynamometers (Maffiuletti et al., 2016).

During the fatiguing exercise protocol participants performed repeated 5-s knee extensor contractions with 5-s of recovery at 85% of their baseline MVIC. Contractions continued until participants failed to reach the required torque in two consecutive contractions. Visual feedback was constantly provided to participants on a large monitor using LabChart software (Version 7.1.3, ADInstruments, Sydney, NSW, Australia). The best MVIC and T_75__ms_ values obtained in the six MVICs before exercise and the first contraction following the exercise protocol (i.e., at fatigue) were used for data analysis. Torque data were recorded using LabChart software on a laptop computer using a 16-bit analog-to-digital converter (PowerLab 16/35, ADInstruments, Sydney, NSW, Australia) sampling at 4000 Hz.

### Surface Electromyography (EMG)

During all contractions surface EMG data were obtained from vastus lateralis (VL) and vastus medialis (VM). EMG electrodes (720 Neuroline, Ambu, NSW, Australia) were placed on the quadriceps muscles as per SENIAM guidelines (Hermens et al., 1999) with a bipolar electrode configuration with a 1-cm inter-electrode distance. Prior to the application of EMG electrodes, the skin was shaved, abraded and cleaned with alcohol to reduce inter-electrode resistance below 5 kΩ. Following this, placement locations were marked with permanent marker ink and the electrodes were applied before being secured using sports cloth tape. EMG electrodes were replaced if they had an inter-electrode resistance above 5 kΩ after DHY as previous research has shown this to provide reliable results (Abbiss et al., 2006). All EMG data were amplified (× 1000) and filtered using a 20–500 Hz band-pass filter before a symmetric root-mean-square filter was applied with a 500-ms averaging window (EMG_RMS_). Prior to analysis EMG_RMS_ data were normalized to the maximal M-wave amplitude (M_max_) to control for potential peripheral changes (Place et al., 2007). EMG data were recorded synchronously with torque data at a 2000 Hz analog-to-digital conversion rate using LabChart software on a laptop computer (PowerLab 16/35, ADInstruments, Sydney, NSW, Australia). The maximal EMG_RMS_ during the six MVICs before fatigue and the first MVIC immediately after fatigue were used for analysis.

### Electrical Stimulation Procedures

Before and 3 h after both DHY and CON the maximal M-wave amplitude (M_max_) and excitation-contraction (E-C) coupling efficiency were assessed. E-C coupling efficiency was also assessed immediately following the fatiguing exercise protocol. Femoral nerve stimulation was used to deliver electrical stimuli for M_max_ assessment, whilst tetanic muscle stimulation was used to assess E-C coupling efficiency. The femoral nerve was located manually and then stimulated using single 0.2-ms square-wave pulses with a constant-current stimulator (DS7A, Digitimer Ltd., Welwyn Garden City, United Kingdom). A compex electrode pen was used to locate the nerve and then an electrode (WhiteSensor 4570M, Ambu, NSW, Australia) was placed on the skin for subsequent stimulations. To find M_max_, resting femoral nerve stimulations were imposed every 10 s from a subthreshold intensity, where no evoked response was observed, until a peak M-wave amplitude was observed. The stimulus intensity used to elicit the M_max_ was then increased by 40% for subsequent testing to ensure a supramaximal stimulus intensity to account for possible depression in motor responses during fatigue (Trajano et al., 2013). To assess E-C coupling efficiency, electrical square-wave stimuli (0.5-ms pulse width) were delivered to the knee extensor muscle belly through four self-adhesive electrodes (5 × 9 cm, Durastick II, Chattanooga group, Hixson, TN, United States) using a constant-current stimulator (DS7A, Digitimer Ltd., Welwyn Garden City, United Kingdom). For all tetanic stimulations, the stimulation intensity necessary to reach 50% of MVIC with a 0.5-s 80 Hz tetanic stimulation was used (Martin et al., 2004). Three evoked contractions of the same duration were delivered with 15 s between each contraction using the following trains: (1) 20 Hz train of 11 pulses (0.05-s interpulse interval); (2) variable-frequency train (VFT) (i.e., 2 pulses at 0.01-s plus, 10 pulses at 0.05-s interpulse interval); (3) 80 Hz train of 36 pulses (0.0125-s interpulse interval) (Trajano et al., 2013). Voluntary activation (VA%), peak twitch torque (T_tw,p_), time to peak twitch (t_tw,p_), peak twitch half relaxation time (t_1/2_), the ratio of torques evoked by 20 and 80 Hz stimulations (20:80), and the ratio of torques 20 Hz and VFT trains (20:VFT) were assessed. The three tetanic trains have been used by previous researchers to provide information relating to muscular calcium concentration, sensitivity and the rate of binding to troponin (Binder-Macleod and Lee, 1996; Martin et al., 2004; Binder-Macleod and Kesar, 2005). The maximal VA% and T_tw,p_ and minimum t_tw,p_ and t_1/2_ during the MVICs and stimulations before fatigue and the first MVIC and stimulation immediately following volitional fatigue were used in data analysis. T_75__ms_, VA%, VL and VM EMG/M were used as markers of central neuromuscular function (Trajano et al., 2013; Maffiuletti et al., 2016) while 20:80, 20:VFT, T_tw,p_, t_tw,p_, and t_1/2_ were used as markers of peripheral neuromuscular function (Behm and St-Pierre, 1997; Behm et al., 2002; Martin et al., 2004). Voluntary activation (VA%) was determined using the correction equation described by Strojnik and Komi (1998):

$$VA\%=100-\text{T}w_{\text{MVIC}}\times (MVIC_{\text{stim}}/MVIC_{\text{peak}})/Tw_{\text{pot}}\times 100$$

where VA% is the corrected voluntary activation, Tw_MV IC_ is the additional twitch torque at MVIC, MVIC_stim_ is the torque level at stimulation, MVIC_peak_ is the peak torque during MVIC, and Tw_pot_ is the potentiated twitch torque at rest.

### Statistical Analysis

Shapiro–Wilk’s tests were used to verify the assumption that the data were normally distributed. Torque, EMG, temperature, blood and urine data were analyzed separately using two-way repeated measures ANOVAs. Data from the POMS-SF and CogState cognitive tests were not normally distributed and were assessed using Friedman’s two-way ANOVAs. Where differences were observed in either normally or non-normally distributed data, *post hoc* tests with the Holm–Bonferroni sequential correction adjustment was used to determine the location of the differences (Holm, 1979). Statistical analysis was performed using SPSS version 24.0 (SPAA Inc., Chicago IL, United States) with statistical significance assumed at *P* < 0.05. Torque and EMG data are reported as mean, standard deviation (SD), confidence intervals (95%) and Hedges’ *g* effect sizes. An effect size of 0.2 was considered small, 0.5 moderate and >0.8 large. All other data are reported as mean (± SD) with *P*-values.

## Results

Body mass was not significantly (*P* = 0.947) different between conditions before intervention (DHY = 80.0 ± 1.9 and CON = 80.3 ± 1.4 kg), however, it was lower immediately after DHY (*P* < 0.001) when compared with CON (77.5 ± 10.4 and 79.7 ± 10.9 kg, respectively). Serum osmolality was significantly (*P* = 0.003) greater immediately following DHY (293.1 ± 4.8 mOsm) than CON (285.1 ± 4.4 mOsm), but no statistical differences were observed at any other time point. Immediately after, haematocrit was greater in DHY than CON (45 ± 3 and 43 ± 2, respectively, *P* = 0.034) but was not different 3 h after. Urine osmolality was greater in DHY (740 ± 249 and 728 ± 326 mOsm) than CON (338 ± 159 and 379 ± 192 mOsm) immediately (*P* < 0.001) and at 3 h post-intervention (*P* < 0.001). Urine specific gravity was greater in DHY (1.027 ± 0.008 and 1.022 ± 0.01 SG) than CON (1.008 ± 0.004 and 1.01 ± 0.005 SG) immediately (*P* < 0.001) and at 3 h post-intervention (*P* < 0.001) (Table 1). Resting heart rate was higher immediately following DHY than CON (117 ± 21 and 56 ± 9, respectively, *P* < 0.001). Both tympanic and core temperature were higher immediately following DHY than CON (*P* < 0.001 for both markers) but were not different at any other timepoint. No significant differences were observed in blood pressure, BDNF or TNFα concentrations between conditions (Table 1).

**Table 1:** Mean (± SD) body mass and cardiovascular, urine and blood markers.

Physiological marker	Condition	Pre-intervention	Immediately	3 h post-
			post-intervention	intervention
Body mass (kg)	DHY	80 ± 10	77 ± 10	79 ± 10
	CON	80 ± 11	80 ± 11***	80 ± 11
Serum osmolality (mOsm/kg)	DHY	286 ± 5	293 ± 5	290 ± 6
	CON	288 ± 4	285 ± 4**	287 ± 5
Haematocrit (%)	DHY	42 ± 2	45 ± 3	43 ± 2
	CON	42 ± 3	43 ± 3*	42 ± 2
Urine osmolality (mOsm/kg)	DHY	448 ± 318	740 ± 249	728 ± 326
	CON	654 ± 321	338 ± 159***	654 ± 321***
Urine specific gravity (SG)	DHY	1.013 ± 0.009	1.027 ± 0.008	1.022 ± 0.01
	CON	1.018 ± 0.01	1.008 ± 0.004***	1.01 ± 0.005***
Resting heart rate (bpm)	DHY	66 ± 10	117 ± 21	68 ± 12
	CON	69 ± 11	56 ± 9***	64 ± 10
Systolic blood pressure (mmHg)	DHY	126 ± 12	122 ± 13	127 ± 11
	CON	125 ± 14	124 ± 14	126 ± 10
Diastolic blood pressure (mmHg)	DHY	69 ± 10	69 ± 7	71 ± 9
	CON	69 ± 9	72 ± 5	69 ± 7
Core temperature (°C)	DHY	37 ± 0.5	39 ± 0.5	37 ± 0.5
	CON	37 ± 0.5	37 ± 0.2***	37 ± 0.2
Tympanic temperature (°C)	DHY	36 ± 0.5	39 ± 0.5	36 ± 0.5
	CON	36 ± 0.5	36 ± 0.5***	36 ± 0.5
Brain-derived neurotropic factor (μg/ml)	DHY	188 ± 149	161 ± 100	106 ± 65
	CON	114 ± 44	202 ± 212	115 ± 115
Tumor necrosis factor alpha (pg/ml)	DHY	0.93 ± 0.23	1.09 ± 0.3	1.02 ± 0.34
	CON	1.13 ± 0.49	1.08 ± 0.32	0.94 ± 0.22

*^∗^*P* < 0.05, ^∗∗^*P* < 0.01, ^∗∗∗^*P* < 0.001 when compared with DHY in at the same time point*.

**FIGURE 1:**
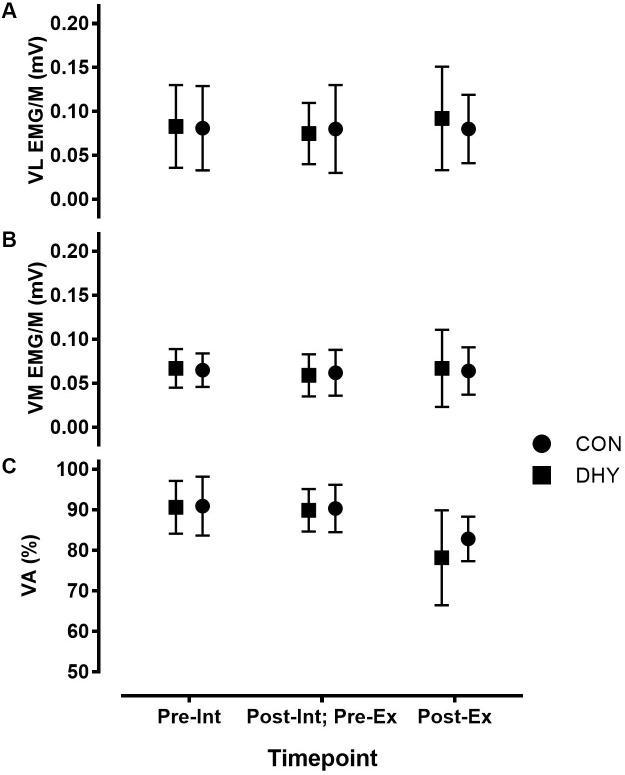
Maximal vastus lateralis (VL) **(A)**, vastus medialis (VM) **(B)** EMG_RMS_/M and voluntary activation (VA%) **(C)** before and 3 h after DHY/CON (Int), as well as before and immediately after fatiguing exercise (Ex). No significant differences were observed between VA (%) or vastus lateralis and medialis EMG_RMS_/M, indicating no influence on central neuromuscular function resulting from acute dehydration.

No significant main effects between conditions were observed for MVIC torque, T_75__ms_, VA% or EMG/M for VL or VM (Table 2 and Figure 1) before or immediately following DHY compared with CON. Furthermore, no significant differences between conditions were observed in contractile properties (T_tw,p_, t_tw,p_, and t_1/2_) or E-C coupling efficiency (20:VFT or 20:80) (Table 2).

**Table 2:** Mean (± SD), 95% confidence limit and Hedges *g* effect size EMG and torque data before and 3 h after DHY and CON.

Neuromuscular	Condition	Pre-intervention	3 h post-	DHY vs. CON	DHY vs.CON
variable			intervention	Pre-intervention	3 h post-intervention
MVIC (Nm)	DHY	295 ± 48	297 ± 49	(-37, 39), 0.02	(-43, 35), 0.08
	CON	296 ± 50	293 ± 52		
T_75__ms_ (Nm)	DHY	74 ± 26	72 ± 24	(-13, 23), 0.324	(-12, 24), 0.214
	CON	79 ± 20	78 ± 22		
20:VFT (Nm)	DHY	0.998 ± 0.04	0.976 ± 0.05	(-0.04, 0.02), 0.291	(-0.01, 0.07), 0.643
	CON	0.986 ± 0.04	1.006 ± 0.04		
20:80 (Nm)	DHY	0.698 ± 0.11	0.705 ± 0.07	(-0.07, 0.08), 0.04	(-0.06, 0.06), 0.013
	CON	0.702 ± 0.09	0.704 ± 0.08		
t_tw,p_ (s)	DHY	0.078 ± 0.002	0.077 ± 0.016	(0.00, 0.01), 0.401	(-0.01, 0.02), 0.258
	CON	0.083 ± 0.017	0.081 ± 0.014		
T_tw,p_ (Nm)	DHY	70 ± 12	71 ± 12	(-8.94, 8.94), 0.000	(-11.32, 7.32), 0.162
	CON	70 ± 11	69 ± 12		
t_1/2_ (s)	DHY	0.074 ± 0.022	0.073 ± 0.021	(-0.02, 0.02), 0.042	(-0.02, 0.01), 0.231
	CON	0.075 ± 0.024	0.068 ± 0.021		

*Differences are displayed as 95% CI (LL, UL) and effect size, no *P*-values are displayed as no significant main effects were observed*.

*Results displayed are maximal voluntary isometric contraction (MVIC), torque at 75 ms (*T*_*75*__*ms*_), 20 Hz:variable frequency train ratio (20:VFT), 20 Hz:80 Hz ratio (20:80), time to peak twitch (t_*tw,p*_), peak twitch torque (*T*_*tw,p*_), peak twitch half relaxation time (t_*1/2*_)*.

Fewer contractions were completed during the fatiguing exercise protocol at 3 h after DHY (17 ± 7) than CON (23 ± 8) [*P* < 0.001, CI (-11.84, -0.16), *g* = 0.775]. MVIC torque and T_75__ms_ decreased after exercise (*P* < 0.001) but this change was not statistically different between conditions (Table 3). Additionally, no significant between-condition effects were observed in VA% or in VL or VM EMG/M before or immediately following fatiguing exercise (Figure 1). No significant differences were observed between conditions in contractile properties (T_tw,p_, t_tw,p_, and t_1/2_) or E-C coupling efficiency (20:VFT or 20:80) in DHY when compared CON (Table 3).

**Table 3:** Mean (± SD), 95% confidence limit and Hedges *g* effect size EMG and torque data before and immediately after fatiguing exercise for both DHY and CON.

Neuromuscular	Condition	Pre-exercise	Post-exercise	DHY vs. CON	DHY vs. CON
variable				Pre-exercise	Post-exercise
MVIC (Nm)	DHY	297 ± 49	236 ± 33	(-43, 35), 0.08	(-31, 15), 0.026
	CON	293 ± 52	228 ± 27		
T_75__ms_ (Nm)	DHY	72 ± 24	58 ± 20	(-12, 24), 0.214	(-18, 14), 0.0
	CON	78 ± 22	56 ± 21		
20:VFT	DHY	0.976 ± 0.05	0.98 ± 0.03	(-0.01, 0.07), 0.643	(-0.04, 0.02), 0.302
	CON	1.006 ± 0.04	0.969 ± 0.04		
20:80	DHY	0.705 ± 0.07	0.678 ± 0.08	(-0.06, 0.06), 0.013	(-0.08, 0.05), 0.194
	CON	0.704 ± 0.08	0.661 ± 0.09		
t_tw,p_ (s)	DHY	0.077 ± 0.016	0.087 ± 0.019	(-0.01, 0.02), 0.258	(-0.02, 0.01), 0.377
	CON	0.081 ± 0.014	0.08 ± 0.017		
T_tw,p_ (Nm)	DHY	71 ± 12	62 ± 11	(-11, 7), 0.162	(-9, 75), 0.211
	CON	69 ± 12	60 ± 7		
t_1/2_ (s)	DHY	0.073 ± 0.021	0.085 ± 0.028	(-0.02, 0.01), 0.231	(-0.02, 0.02), 0.104
	CON	0.068 ± 0.021	0.088 ± 0.028		

*Differences are displayed as 95% CI (LL, UL) and effect size, no *P*-values are displayed as no significant main effects were observed*.

*Results displayed are maximal voluntary isometric contraction (MVIC), torque at 75 ms (*T*_*75*__*ms*_), 20 Hz:variable frequency train ratio (20:VFT), 20 Hz:80 Hz ratio (20:80), time to peak twitch (t_*tw,p*_), peak twitch torque (*T*_*tw,p*_), peak twitch half relaxation time (t_*1/2*_)*.

Perception of tension was greater immediately after DHY when compared with CON (10 ± 5 and 7 ± 1, respectively; *P* = 0.036) but was not different at any other time point. Depression (13 ± 6 and 8 ± 1, respectively; *P* = 0.033) and anger (13 ± 8 and 7 ± 1, receptively, *P* = 0.036) were only greater immediately after DHY when compared with CON. Confusion was also greater only immediately after the DHY when compared with CON (10 ± 5 and 6 ± 3, respectively; *P* = 0.045). However, perception of fatigue after DHY was greater than CON immediately (17 ± 6 and 6 ± 2, respectively; *P* = 0.003) and 3 h after (11 ± 5 and 6 ± 2, respectively; *P* = 0.012). No main effects were observed in vigor at any time point (Table 4).

**Table 4:** Mean (± SD) mood state before and immediately and 3 h post-intervention.

Mood state	Condition	Pre-	Immediately	3 h post-
		intervention	post-	intervention
			intervention	
Tension	DHY	8 ± 2	10 ± 5	8 ± 4
	CON	7 ± 2	7 ± 1*	7 ± 2
Depression	DHY	9 ± 2	13 ± 6	11 ± 5
	CON	9 ± 2	8 ± 1*	8 ± 0.5
Anger	DHY	8 ± 2	13 ± 8	9 ± 4
	CON	8 ± 2	7 ± 1*	8 ± 1
Fatigue	DHY	7 ± 3	17 ± 6	11 ± 5
	CON	7 ± 4	6 ± 2**	6 ± 2*
Confusion	DHY	7 ± 4	10 ± 5	8 ± 3
	CON	7 ± 5	6 ± 3*	6 ± 2
Vigor	DHY	16 ± 5	11 ± 4	13 ± 6
	CON	16 ± 6	13 ± 5	13 ± 5

*^∗^P < 0.05, ^∗∗^P < 0.01 when compared with DHY in at the same time point*.

The majority of cognitive function measures were unchanged except for one-card accuracy and speed. One card learning accuracy was significantly (*P* = 0.02) less immediately following DHY compared with CON (68 ± 9 and 75 ± 8%, respectively) but no differences were observed at 3 h (Table 5). Additionally, one card learning speed immediately following DHY was significantly (*P* = 0.048) faster than CON (865 ± 192 and 909 ± 184 ms, respectively) but no differences were observed after 3 h (Table 5).

**Table 5:** Mean (± SD) cognitive marker immediately and 3 h post-intervention.

Cognitive assessment	Condition	Immediately	3 h post-
		post-	intervention
		intervention	
Detection – speed (ms)	DHY	343 ± 64	363 ± 64
	CON	359 ± 90	353 ± 74
Detection – accuracy (%)	DHY	97 ± 3	95 ± 6
	CON	96 ± 3	97 ± 2
Identification – speed (ms)	DHY	536 ± 118	544 ± 112
	CON	518 ± 92	522 ± 115
Identification – accuracy (%)	DHY	96 ± 3	94 ± 7
	CON	96 ± 4	95 ± 7
One card learning – speed (ms)	DHY	865 ± 192	918 ± 224
	CON	909 ± 184*	871 ± 194
One card learning – accuracy (%)	DHY	68 ± 9	70 ± 14
	CON	75 ± 8*	74 ± 9
One back – speed (ms)	DHY	724 ± 148	703 ± 172
	CON	703 ± 172	724 ± 148
One back- accuracy (%)	DHY	90 ± 8	94 ± 5
	CON	94 ± 6	95 ± 4
Maze errors (n)	DHY	45 ± 16	49 ± 27
	CON	46 ± 17	46 ± 14

*^∗^*P* < 0.05 when compared with DHY in at the same time point*.

## Discussion

In the present study, the influence of the acute loss of 3% of body mass, achieved by 3 h of passive dehydration in the heat, on muscular strength and endurance, central and peripheral neuromuscular function, psychological profile, cognitive function, body temperature and markers of hydration status was examined. While previous research has examined the influence of acute dehydration on muscular performance, a novel aspect of the present study was investigating the central and peripheral neuromuscular mechanisms underpinning such performance. The main observations were that: (i) 3 h of *ad libitum* fluid and food consumption following acute dehydration of 3% resulted in the recovery of body mass, serum osmolality and haematocrit but not urinary markers of hydration status, (ii) the number of isometric knee extensor contractions performed at 85% of MVIC was less 3 h after DHY when compared with CON, (iii) peak torque or T_75__ms_ were not significantly different between conditions at any time point, (iv) there were no significant differences between DHY and CON conditions in markers of either central and peripheral neuromuscular function at any time point, and (v) acute dehydration increased perception of fatigue both immediately and 3 h after DHY when compared with CON.

As anticipated, participants in this study lost more body mass during DHY (3.2% of body mass) than CON (0.4%), with the decrease during DHY being similar to that previously observed prior to competition in combat sports (Barley et al., 2017a). Physiological markers of hydration status (USG, urine osmolality, haematocrit, serum osmolality and body mass) indicated a greater level of dehydration immediately after DHY than CON (Table 1). *Ad libitum* fluid and food consumption resulted in body mass, serum osmolality and haematocrit returning to baseline, however, urine osmolality and USG did not (Table 1). These results are similar to those reported previously in research examining acute dehydration in combat athletes (Barley et al., 2017b) and indicate the complexity of assessing dehydration and the limitations of using a single (or even multiple) measure of hydration status (Armstrong, 2007). Nevertheless, and of significant practical importance, acute dehydration followed by a rehydration period has been found to impair exercise performance even when several markers of hydration have returned to baseline (Barley et al., 2017b).

While studies have indicated that dehydration may (Minshull and James, 2012; Rodrigues et al., 2014) or may not (Cheuvront et al., 2006; Kraft et al., 2012) compromise anaerobic performance, few studies have examined measures of central and peripheral neuromuscular function and performance following rehydration. In the present study we observed no differences in maximal strength between conditions at any timepoint (Tables 2, 3). However, we did observe a decrease in muscular strength-endurance 3 h after the DHY protocol, as evidenced by a 28% decrease in the number of contractions performed at 85% of MVIC (*g* = 0.775). It is important to consider that while there were no differences between conditions in maximum MVIC torque or T_75__ms_ immediately post-exercise, less total work (contractions at 85% MVIC) was completed after DHY than CON. Thus, the ability to generate maximal force was impaired after DHY because the loss of force generating capacity was the same after DHY and CON despite fewer contractions being performed. The lack of change in maximal strength alongside the decrease in muscular endurance supports the findings of previous research (Montain et al., 1998; Bigard et al., 2001). Such results suggest that the use of acute dehydration weight loss strategies in weight-restricted sports can impair physical performance even up to 3 h after the intervention and therefore may not be an optimal strategy for competitive performance. These results may also be applicable to occupational or military settings where dehydration is likely to occur during normal operational duties. Indeed, it appears that in such settings *ad libitum* rehydration may be effective in stabilizing several markers of hydration, but some markers of hydration and aspects of physical function might also remain compromised for at least 3 h.

Despite the negative influence of dehydration on muscular strength-endurance, no changes in markers of central or peripheral neuromuscular function were observed. Indeed, no differences in central drive (i.e., T_75__ms_, VA% or VL or VM EMG/M; Tables 2, 3 and Figure 1) were observed 3 h after DHY or CON or immediately following the fatiguing exercise. Likewise, no differences in peripheral neuromuscular function (i.e., T_tw,p_, t_tw,p_ or t_1/2_; Tables 2, 3) were observed between conditions at any time point. This aligns with previous research which has shown no change in the neuromuscular function of hypohydrated athletes (Evetovich et al., 2002). However, the present study contributes to the body of research by demonstrating that the lack of neuromuscular changes remain persistent after 3 h. These results differ from previous studies investigating the influence of thermal exposure on neuromuscular fatigue (Ross et al., 2011; Goodall et al., 2015), however, the present study did not measure neuromuscular function during thermal stress but instead following a recovery period. Additionally, no differences in TNAα were observed at any timepoint indicating a lack of DHY-induced tissue damage. While DHY did not appear to influence E-C coupling processes, we did observe a non-significant moderate effect (*g* = 0.643) in the 20:VFT 3 h after DHY but not following fatiguing exercise (Table 2), potentially indicating a decrease in calcium sensitivity 3 h after DHY (Binder-Macleod and Kesar, 2005; Nielsen, 2009). It is important to consider that we did not measure all components of neuromuscular function [e.g., motoneuron facilitation (Heckman et al., 2005; Heckman and Enoka, 2012)] so it is possible that some changes exist that may partly explain the loss of function. Nevertheless, the lack of effect of DHY on RFD, EMG/M and VA% (central drive) or in twitch properties or tetanic torques (peripheral function) strongly indicate a lack of DHY-induced decrease in neuromuscular function. Alternatively, other non-neural mechanisms such as an elevated core temperature, reduction in muscle glycogen or impaired cardiovascular function may explain the impairment in muscular performance but the lack of difference in thermal or cardiovascular markers (Table 1) alongside the nature of the fatiguing exercise used in the present study makes such explanations unlikely (González-Alonso et al., 1997; Casa, 1999; Barley et al., 2017b). In addition, previous research investigating the mechanisms behind acute dehydration induced fatigue has found it is not the result of H ^+^ or P_1_ concentration (Montain et al., 1998). Clearly, further research is needed to determine the mechanisms responsible for the impairment in exercise performance resulting from acute DHY.

Another possibility is that an increase in mental fatigue (e.g., A psychobiological effect characterized by subjective feelings of “tiredness and “lack of energy”) may explain the observed decrease in muscular strength-endurance (Marcora et al., 2009). Indeed, mental fatigue has been previously linked to decrements in exercise performance despite markers of central fatigue (i.e., central motor drive specifically) remaining unchanged (Marcora et al., 2009; Pageaux et al., 2015). Consistent with the hypothesis of mental fatigue influencing performance, the present results show that perceptions of tension, depression, anger, fatigue and confusion were all greater than CON immediately after DHY, however, only the perception of fatigue remained greater than CON after 3 h (Table 4). Therefore, it seems plausible that altered fatigue perception contributed to the decreased number in knee extensor contractions performed during fatiguing exercise (Marcora et al., 2009; Tucker, 2009). These results are consistent with previous research examining the effects of lower levels of dehydration (<2%) and the combination of dehydration and food restriction on mood (Hall and Lane, 2001). In addition to the change in mood we also found evidence of compromised cognitive function after DHY. Indeed, decision-making time and accuracy in one card learning were both reduced immediately after DHY (Table 5); however, these were not different at 3 h post-intervention. While previous research has linked decreased fluid ingestion and hyperthermia to changes in blood BDNF concentrations and cognitive function (Roh et al., 2017), we observed no differences in blood BDNF at any time point although there was a large inter-subject variability in the results throughout the study which limits what interpretations can be made from the data (Table 1). Therefore it is plausible that the decreases in cognitive function observed in this study did not result solely from physiological changes but rather an influence of mental fatigue on task engagement (Van der Linden et al., 2003). Further research that takes a system biological approach to investigate the potential for mental fatigue to contribute to the performance decrements resulting from acute dehydration is required.

We report that acute heat-induced dehydration of 3% body mass impairs muscular strength-endurance and increases perception of fatigue without detectibly influencing markers of central and peripheral neuromuscular function at 3 h after the weight loss intervention despite *ad libitum* consumption of food and fluids. Such findings potentially indicate that an increased mental fatigue resulting from acute dehydration, which persists even after fluid and food consumption are resumed, influenced the muscle work achievable before “fatigue.” In addition, we provide evidence suggesting that athletes may not achieve adequate rehydration when allowed *ad libitum* fluid and food consumption following weight loss, as evidenced by a greater USG and urine osmolality 3 h after. A strength of this study was that a wide range of scientifically valid markers of central and peripheral neuromuscular function were utilized following a highly controlled dehydration protocol. However, the present study intentionally did not standardize food and fluid consumption during the recovery period to maximize ecological validity at the cost of developing a greater understanding of how recovery practices following acute dehydration may influence exercise performance. These findings outline the need for further research into the mechanisms by which acute dehydration impairs exercise performance, specifically regarding the relationship between acute dehydration and mental fatigue.

## Author Contributions

OB, CA, and DC conceived and designed the study. OB performed the experiments and analyzed the data. OB, CA, DC and AB interpreted the results of experiments. OB and CA drafted the manuscript. All authors edited and revised the manuscript before approving the final version.

## Conflict of Interest Statement

The authors declare that the research was conducted in the absence of any commercial or financial relationships that could be construed as a potential conflict of interest.
